# Blastic plasmacytoid dendritic cell neoplasm of skin, a rare dermatohematologic malignancy—A case report

**DOI:** 10.1002/ccr3.9398

**Published:** 2024-08-28

**Authors:** Ramtin Edjtemaei, Alireza Ghanadan, Fereshte Ameli

**Affiliations:** ^1^ Department of Pathology, School of Medicine, Razi Hospital Guilan University of Medical Sciences Rasht Iran; ^2^ Department of Pathology, School of Medicine, Imam Khomeini Hospital Tehran University of Medical Sciences Tehran Iran

**Keywords:** Blastic plasmacytoid dendritic cell neoplasm, BPDCN, hematologic malignancy, skin cancer

## Abstract

**Key Clinical Message:**

Blastic plasmacytoid dendritic cell neoplasm is a rare hematologic malignancy and appropriate diagnosis encounters difficulties in both clinical and pathologic aspects. This case report aims to present a clinical case to help familiar clinicians and pathologists with this rare entity.

**Abstract:**

Blastic plasmacytoid dendritic cell neoplasm (BPDCN) is an uncommon hematologic malignancy. Because of the rarity of the disease and aggressive behavior, we present this case. A 71‐year‐old man presented with a forehead ulcerated skin lesion. On histopathologic examination, pan‐dermal atypical mononuclear infiltrate, consisting of small‐medium sized cells with fine chromatin pattern was seen without epidermotropism which were immunoreactive for CD123, CD56, TdT and CD4, while negative for CD3, CD20, and MPO that confirmed the diagnosis of BPDCN. BPDCN is a highly aggressive hematologic malignancy derived from plasmacytoid dendritic cells. Male‐to‐female ratio is 3.3:1. Skin involvement can present as either an isolated purplish nodule or disseminated purplish nodules or papules or macules. On microscopic examination, skin involvement is characterized by monomorphic infiltrates of immature neoplastic cells with blastoid morphology, involving the superficial and deep dermis, often with extension into the subcutis with epidermal spare. Immunophenotyping shows usually positive reactions for CD123, CD45, CD4, CD56, TCL1, CD2AP, CD43, BCL2, TdT, Granzyme B, and TCF4, whereas tumor cells are negative for CD3, CD19, CD20, MPO, CD13 and Lysozyme. Differential diagnoses of BPDCN include myeloid sarcoma, myelomonocytic leukemia, mature plasmacytoid dendritic cell proliferation (MPDCP) and Merkel cell carcinoma. Pathologists ought to be familiar with this WHO entity for early disease diagnosis, because of disease rarity and diagnosis difficulties.

## 1 INTRODUCTION

Blastic plasmacytoid dendritic cell neoplasm (BPDCN) is an uncommon hematologic malignancy originating from plasmacytoid dendritic cells (PDCs).^1^ These cells harbor a specific immunophenotype, in which tumor cells are positive for CD4 and CD56 and negative for CD3 and CD19.^2^ The BPDCN is classified as a separate category besides acute myeloid leukemia and B/T cell leukemia/lymphoma in the 2022 World Health Organization Classification of Hematolymphoid Tumors 5th edition.^1^ It commonly involves the skin, and skin lesions including nodules and papules can be the only disease presentation; however, it can involve the bone marrow, lymph nodes and spleen.^1^, ^3^, ^4^ The disease presentation varies from isolated purplish skin nodules to disseminated bruise‐like papules. The latter is the most characteristic clinical presentation of the disease. Lymph node involvement occurs in 20% of cases.^1^ A leukemic reaction may also occur. Breast tissue infiltration by BPDCN has been reported in one case.^4^ Following the initial response to chemotherapy, relapse occurs in most cases with the involvement of skin and/or other organs including CNS.^1^ The aggressive course and poor prognosis of the patients as well as diagnosis difficulties for pathologists and clinicians are problems that have not been resolved so far. Here we describe a case of BPDCN, someone who presented with a forehead skin lesion. Immunophenotyping by immunohistochemistry methods confirmed the diagnosis.

## 2 PATIENT INFORMATION

The present case has been reported in line with the SCARE criteria.^5^

A 71‐year‐old man presented with a forehead skin lesion from 2 months ago that immediately appeared after a traumatic injury. The patient had no previous history of medical diseases. No other skin lesions were found on physical examination and the patient had not received any medical treatment before.

## 3 CLINICAL FINDINGS

**FIGURE 1:**
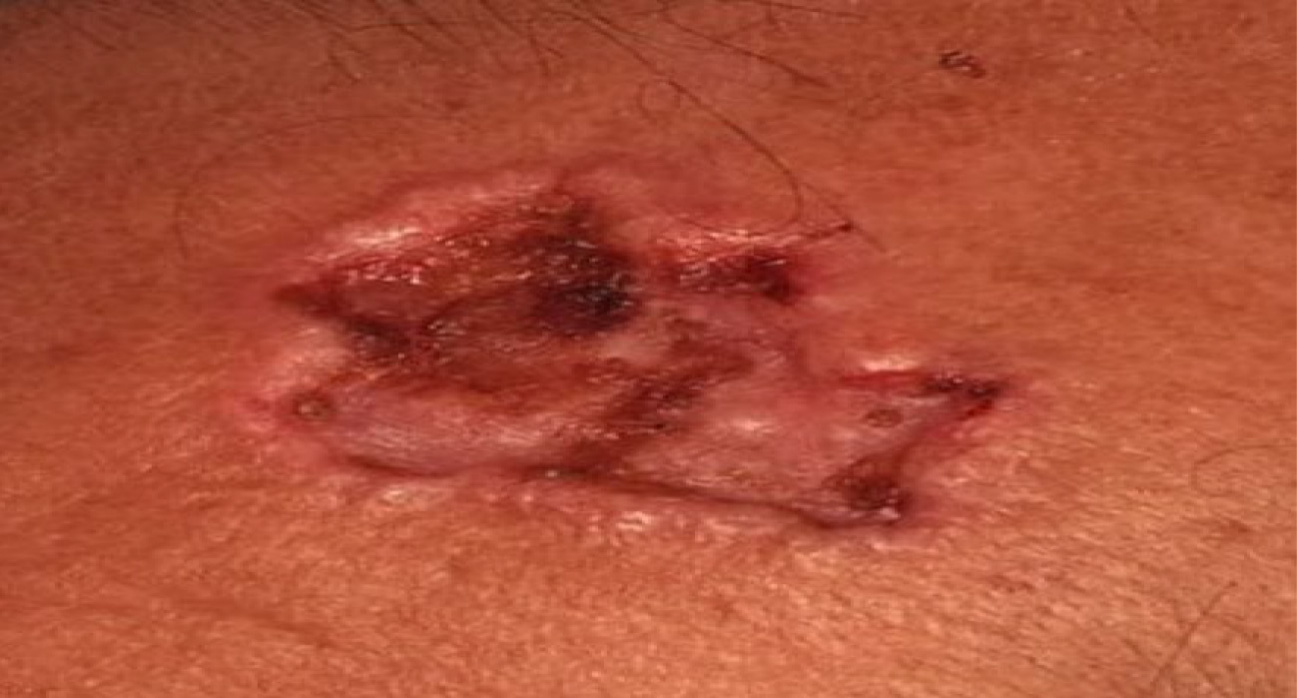
Gross appearance of patient's forehead skin lesion.

**FIGURE 2:**
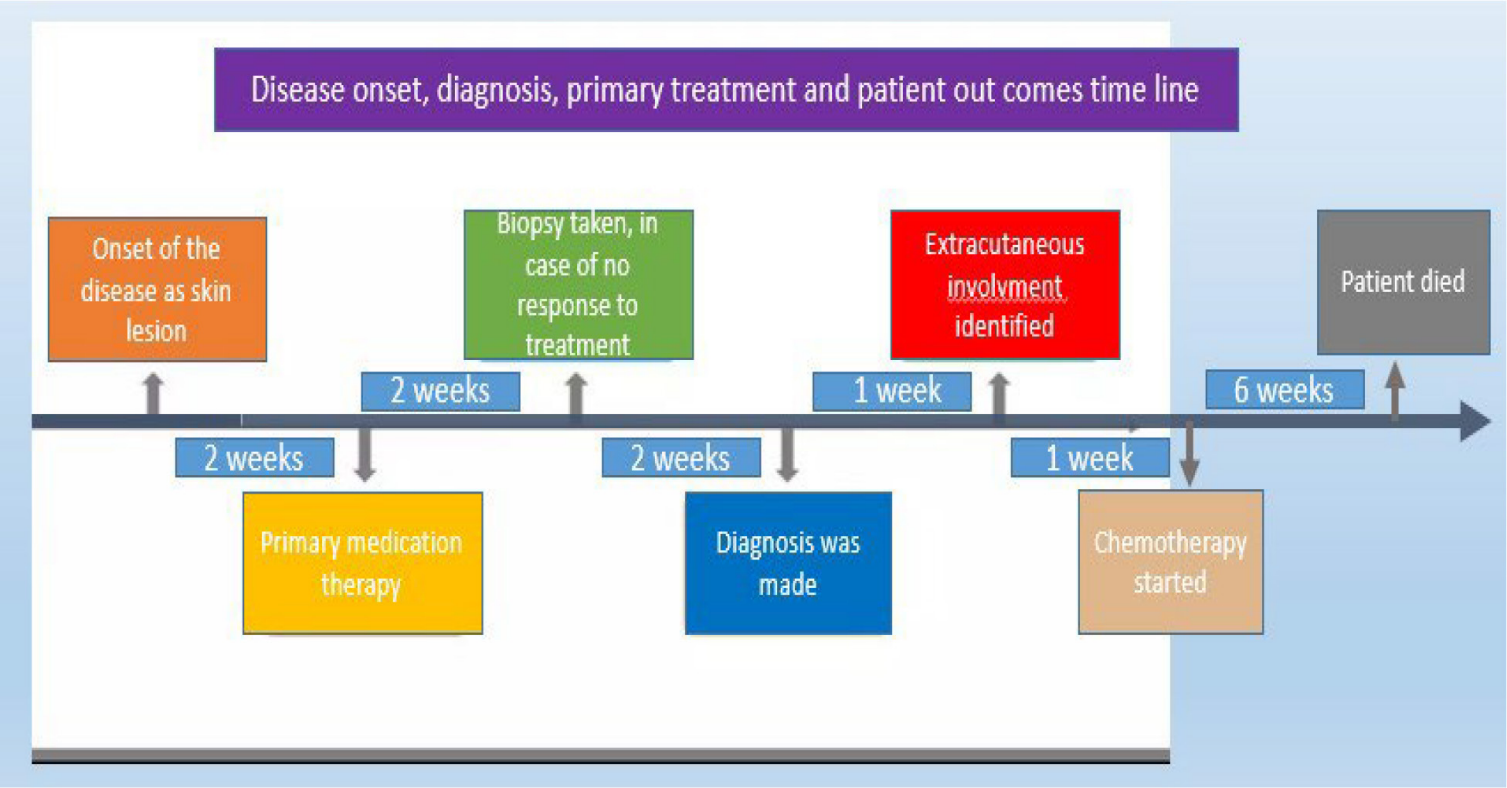
Timeline demonstrating disease onset, diagnosis and patient outcomes.

The lesion was a crusted erythematous ulcerated skin plaque with slightly elevated borders measuring 2.5 cm in length on the forehead skin (Figure 1). Although physical examination revealed no hepatosplenomegaly, further evaluations revealed periportal, and perihepatic lymphadenopathy and bilateral pleural effusion (Figure 2). The CBC test was rather unremarkable, despite mild lymphocytosis (absolute lymphocyte count of 4500/μl). Bone marrow aspiration/biopsy (at another center) revealed a normal population of hematopoietic cells without obvious evidence of bone marrow involvement. Brain computed tomography (CT) revealed no evidence of CNS involvement.

## 4 DIAGNOSTIC ASSESSMENT

**FIGURE 3:**
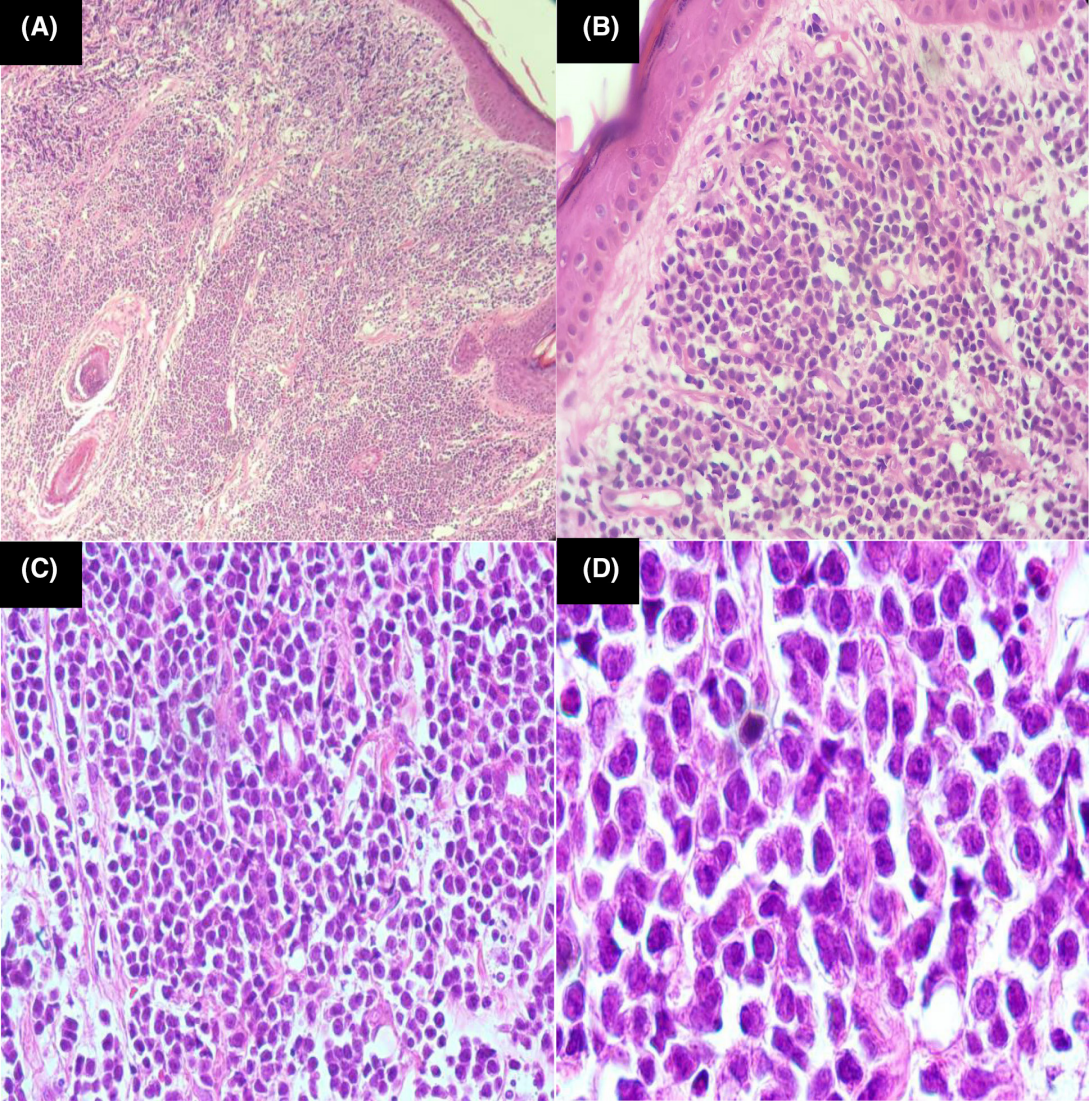
Histopathologic evaluation shows dense and monomorphic infiltration of atypical large‐sized blast‐like cells in the dermis with epidermal spare. Higher magnification (D) shows fine chromatin and small nucleoli in tumor cells. (Magnification: A: 40×, B: 100×, C: 400×, D:1000×).

The patient underwent incisional biopsy and the sample was sent for histopathologic evaluation. The histopathologic examination revealed skin tissue with dense and diffuse, pan‐dermal infiltrate, composed of small‐medium sized cells with high nuclei/cytoplasmic (N/C) ratio, and fine chromatin pattern. The infiltrate extended to the deep dermis and dissected collagen bundles. The overlying epidermis was separated from tumor cells by a Grenz zone of normal collagen. No epidermotropism was identified (Figure 3A–D).

**FIGURE 4:**
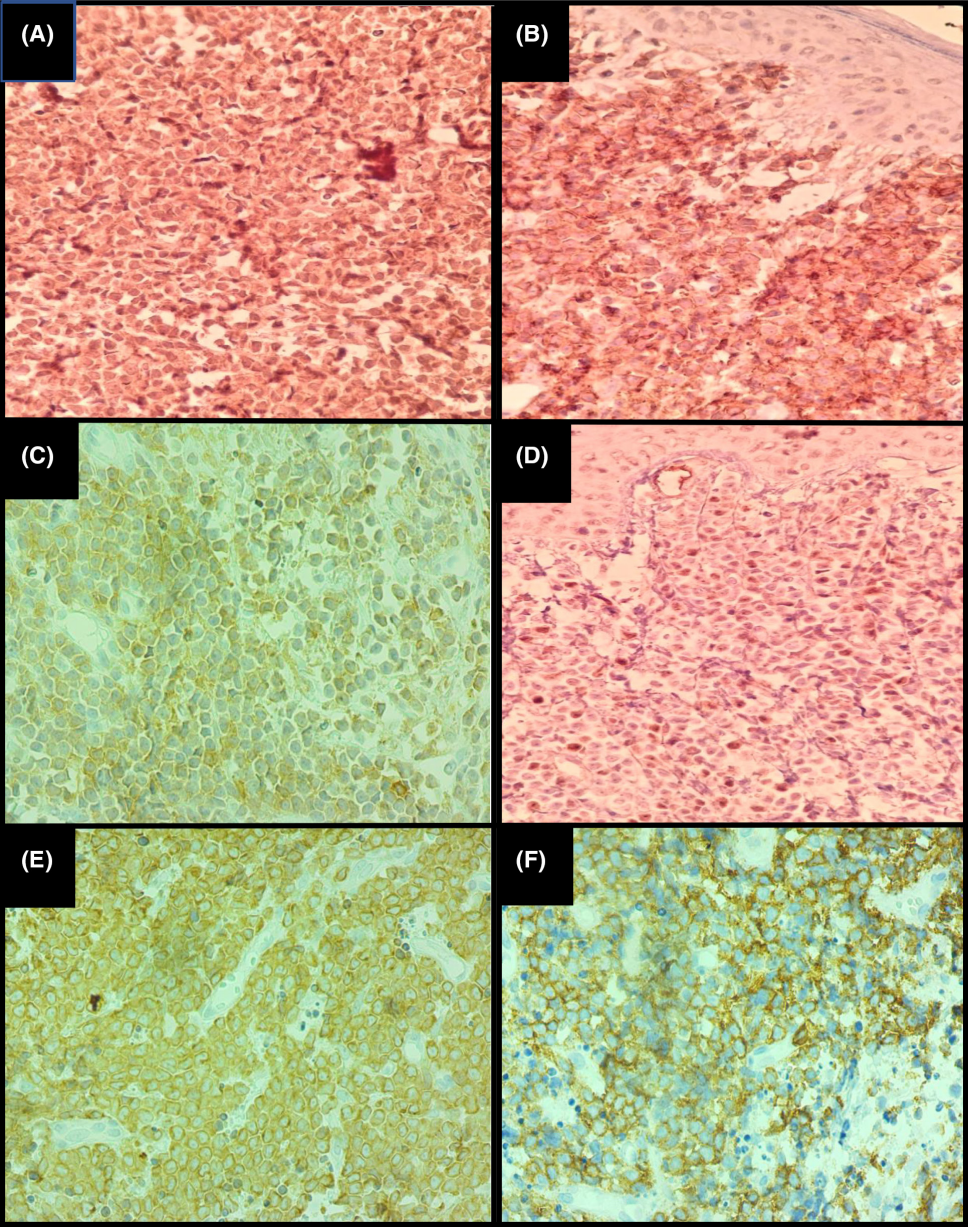
Immunophenotyping of the skin tumor; positive immunoreactivity of tumor cells for CD123 (A), CD56 (B), CD4 (C), TdT (D), BCL2 (E), and CD43 (F).

**FIGURE 5:**
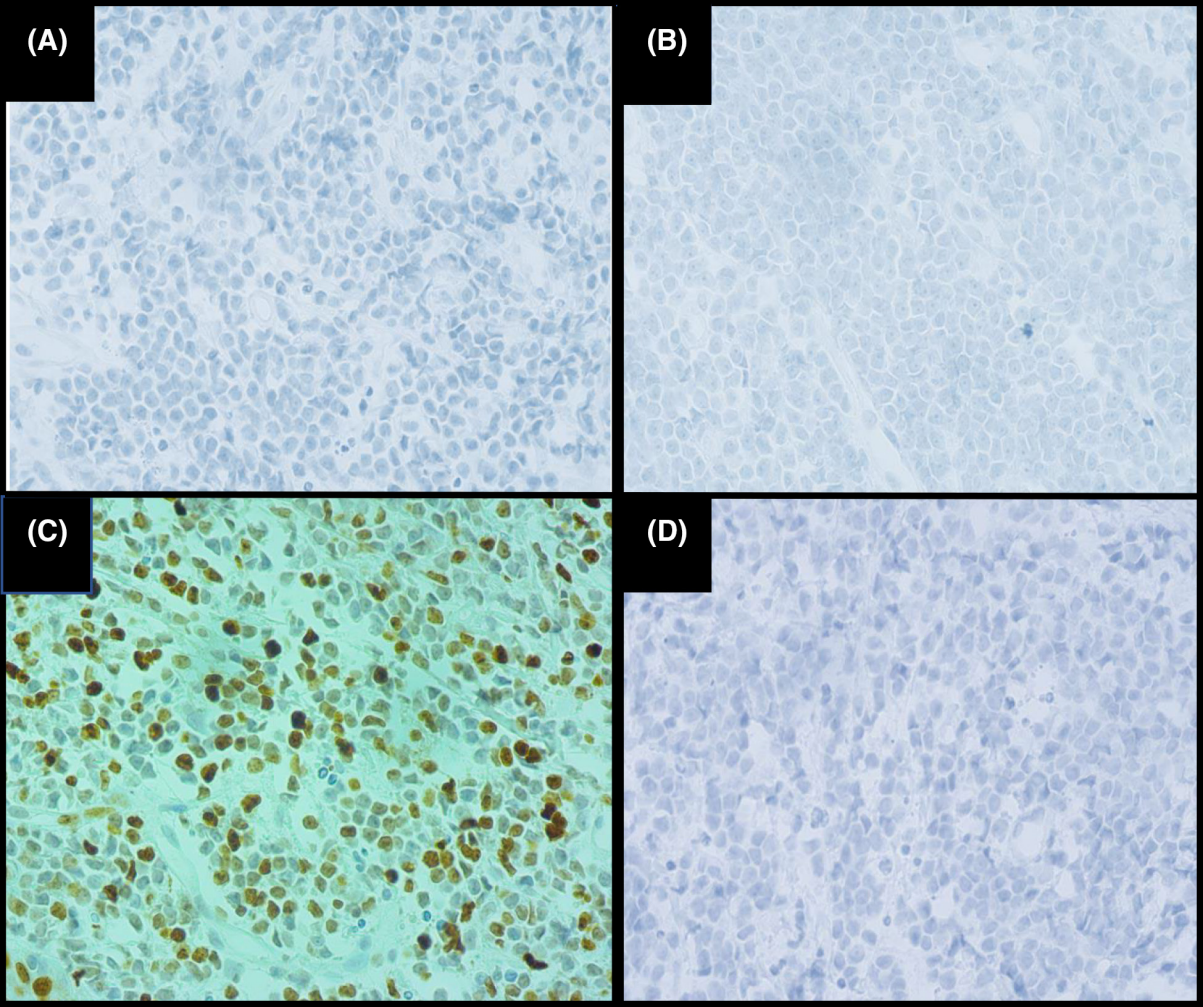
Immunophenotyping of the skin tumor; negative immunoreactivity of tumor cells for CD3 (A), CD20 (B) and MPO (C, Ki67 proliferation index (D) shows 40%–45% nuclear staining.

In immunohistochemistry, tumor cells diffusely expressed CD45, CD56, CD4, BCL2 TdT and CD43. (Figure 4A–F). The neoplastic cells showed negative immunoreactivity for CD20, CD3, MPO, CD34, and synaptophysin. Ki67 revealed proliferative activity in about 40%–45% of tumor cells (Figure 5A–D).

These findings confirmed the diagnosis of BPDCN.

## 5 THERAPEUTIC INTERVENTION

Primary topical medication therapy was unsuccessful and therefore incisional biopsy of the lesion was performed. ALL‐type induction chemotherapy with Daunorubicin/Cytarabine was performed, 2 weeks after the histopathologic diagnosis of BPDCN was made.

## 6 FOLLOW‐UP AND OUTCOMES

The disease progression was markedly rapid. Unfortunately, his health status rapidly deteriorated. The patient died in the background of chemotherapy complications and bradycardia, 2 months after diagnosis, soon after chemotherapy started.

## 7 DISCUSSION

BPDCN, also known as Blastic NK‐cell lymphoma, is a highly aggressive hematologic malignancy derived from PDCs. The male‐to‐female ratio is 3.3:1. The mean age at diagnosis is 61–67 years, but the disease occurs at any age, including young infants. The disease has a significant propensity for skin involvement.^2^ Other common sites include the bone marrow and lymph nodes.^3^

Skin involvement can present as isolated purplish nodules or disseminated papules or macules.^1^ Some patients present without skin involvement and show leukemic involvement of peripheral blood and marrow spaces.^3^ Central nervous system (CNS) infiltration can rarely happen, with a tendency to occur as relapse.^6^ CNS involvement in BPDCN also presents with neurological symptoms and makes diagnosis and treatment more challenging.^7^ The median survival in BPDCN is 10–19 months. Commonly relapse occurs after initial treatment response.

On microscopic examination, BPDCN is characterized by a dermal infiltrate of small to medium‐sized blast‐like cells. The nucleus shows blastoid features with open chromatin and a single or multiple prominent nucleoli. The cytoplasm is usually scant and is agranular grayish‐blue.^3^

Skin involvement is characterized by monomorphic infiltrates of immature neoplastic cells involving the superficial and deep dermis, often with extension into the subcutis.^4^ The epidermis is typically not involved. Mitotic figures are frequently available. Adnexal components are usually uninvolved.^3^

The brownish color in immunohistochemistry slides indicates a positive reaction for each marker and would have a cytoplasmic or nuclear pattern of staining. Immunophenotyping usually shows positive reaction for CD123, CD45, CD4, CD56, TCL1,^5^ CD2AP, CD43, BCL2, TdT, Granzyme B and TCF4, while negative results for CD3, CD19, CD20, MPO, CD13 and Lysozyme. Positive reaction for S100 protein may occur in 25%–30% of cases.^4^ Other mentioned positive markers in literature include BDCA‐2 (blood dendritic cell antigen‐2) and CD303.^8^ BPDCN is not associated with EBV.^3^, ^9^

Cytogenetic studies in BPDCN have been performed and show abnormal karyotype in two‐thirds of the cases with involvement of 5q21 (in 72% of cases), 12p13 (in 64% of cases), 13q13‐21 (in 64% of cases), 6q23‐qter (in 50% of cases), 15q (in 43% of cases), and loss of chromosome 9 (in 28% of cases). Unfortunately, none of these cytogenetic alterations are specific to the BPDCN.^10^

Differential diagnoses of BPDCN include myeloid sarcoma, myelomonocytic leukemia (which can express CD4, CD56 and CD123), mature plasmacytoid dendritic cell proliferation (MPDCP) and Merkel cell carcinoma (MCC) which are all excluded in this case by appropriate immunohistochemistry study. The presence of myelomonocytic specific markers, such as CD13, CD15, CD33, myeloperoxidase (MPO), CD14, and CD64 is useful for establishing the diagnosis of CD56‐positive acute myeloid leukemia, first presenting in the skin.^11^ MPDCP is a premalignant state with the potential to become malignant in the form of BPDCN or other kinds of myeloid malignancies.^12^ MPDCPs consist of aggregates of bland‐looking PDCs. The PDCs in these aggregates have the same phenotype as their normal counterparts, although aberrant expression of CD2, CD5, CD7, CD10, CD13, CD14, CD15, and/or CD33 may occur. To differentiate from BPDCN, CD56 is negative in most cases of MPDCPs or shows focal and weak reactivity.^13^ A low Ki67 proliferation index (less than 10%) and negative reaction for TdT are also helpful.^3^ Rather than that, increased expression of genes involved in NOTCH signaling and BCL2, as well as aberrant activation of the NF‐kappa B pathway, occurs in BPDCN, but not in normal PDCs.^10^ Extranodal NK/ T cell lymphoma is a lymphoid neoplasm that typically involves the nose, nasopharynx, oropharynx, the waldeyer's ring, and the upper airways. The neoplasm is an EBV‐associated lymphoma characterized by destructive angiocentric lymphoid infiltration which shows immunoreactivity for CD3, CD56, and Granzyme.^14^, ^15^ Skin is hardly involved in this neoplasm. Zonal necrosis is present. Our case shows negative immunoreactivity for CD3 and no evidence of angiodestructive lesion is detected. MCC is a primary skin malignancy. The tumor cells are small blue round cells with uniform round to oval nuclei, inconspicuous nucleoli and scant cytoplasm. Characteristically, the majority of MCCs express cytokeratins, CD56 and CK20, but are negative for CD45.^16^ Our case revealed negative results for cytokeratin.

The optimal treatment of BPDCN is not well understood. It is mentioned that more intensive, acute leukemia–like induction ALL‐type regimens (in comparison with AML‐or lymphoma‐type regimens) followed by hematopoietic stem cell transplantation are associated with better outcomes in BPDCN.^6^, ^17^, ^18^ Food and Drug Organization (FDA) approved anti CD123‐targeting agents such as Tagraxofusp in 2018.^6^, ^19^ CNS prophylaxis by intrathecal chemotherapy is performed in some cases where the chemotherapy regimens do not include CNS coverage.^6^ In a study performed by Bekkenk et al in 2004, younger patients (less than 40 years of age) who presented with skin‐limited disease underwent aggressive acute leukemia treatment and had a more favorable prognosis. High TdT expression is also associated with better outcomes.^20^ Hematopoietic‐cell transplantation is recommended for adults in first complete remission.^21^

## 8 CONCLUSION

In the present paper, we reported a rare skin hematologic malignancy with the diagnosis of BPDCN, which desirably involves skin as nodules, papules, or macules. Pathologists and medical oncologists ought to be familiar with this WHO entity for early disease diagnosis, because of disease rarity and diagnosis difficulties. Histomorphologic patterns of BPDCN, immunophenotyping and some of the mimickers of the disease are described in the presented case report. We hope that this case report and future studies help determine early and accurate diagnostic and therapeutic strategies for this rare skin hematologic malignancy.

## AUTHOR CONTRIBUTIONS

**Ramtin Edjtemaei:** Conceptualization; data curation; project administration; supervision; writing – original draft. **Alireza Ghanadan:** Conceptualization; supervision; validation. **Fereshte Ameli:** Conceptualization; supervision; validation.

## FUNDING INFORMATION

The authors received no financial support for the research, authorship, and/or publication of this article.

## CONFLICT OF INTEREST STATEMENT

All authors declared that there are no conflicts of interest.

## ETHICS STATEMENT

All procedures were performed under the 1964 Helsinki Declaration and its latest amendments. Informed written consent was obtained from the patient's offspring (the patient's son, since the patient was not alive when performing this case report). Patient's personal information including identity and face picture were never published anywhere.

## CONSENT

Written informed consent was obtained from the patient under the journal's patient consent policy.

## Data Availability

The data that support the findings of this study are available from the corresponding author upon reasonable request.

## References

[1] Li W . The 5th edition of the World Health Organization classification of hematolymphoid tumors. Exon Publications. 2022;1‐21. 36395314

[2] Saadeh D , Kurban M , Abbas O . Plasmacytoid dendritic cell role in cutaneous malignancies. J Dermatol Sci. 2016;83(1):3‐9. 27236509 10.1016/j.jdermsci.2016.05.008

[3] Bruneau J , Molina TJ . WHO classification of tumors of hematopoietic and lymphoid tissues. Hema. 2020;501‐505.

[4] Döger FK , Cetin ED , Hekimgil M , et al. Plasmacytoid dendritic cell tumor: A case report/Plasmasitoid dendritik hücreli tumor: Bir olgu sunumu. Turk J Haematol. 2011;28(4):312‐316. 27264589 10.5152/tjh.2011.87

[5] Sohrabi C , Mathew G , Maria N , Kerwan A , Franchi T , Agha RA . The SCARE 2023 guideline: updating consensus surgical CAse REport (SCARE) guidelines. Int J Surg Lond Engl. 2023;109(5):1136‐1140. 10.1097/JS9.0000000000000373 PMC10389401 37013953

[6] Haddadin M , Taylor J . Chemotherapy options for blastic plasmacytoid dendritic cell neoplasm. Hematology/Oncology Clinics. 2020;34(3):539‐552. 10.1016/j.hoc.2020.01.011 32336418

[7] Pemmaraju N , Wilson NR , Khoury JD , et al. Central nervous system involvement in blastic plasmacytoid dendritic cell neoplasm. Blood, the J Am Society Hematol. 2021;138(15):1373‐1377. 10.1182/blood.2021011817 34098573

[8] Herling M , Teitell MA , Shen RR , Medeiros LJ , Jones D . TCL1 expression in plasmacytoid dendritic cells (DC2s) and the related CD4+ CD56+ blastic tumors of skin. Blood. 2003;101(12):5007‐5009. 12576313 10.1182/blood-2002-10-3297

[9] Cui X‐B , Jin J , Pang X‐L , et al. A case of blastic plasmacytoid dendritic cell neoplasm with ecchymotic lesions on the whole body. Int J Clin Exp Pathol. 2014;7(7):4391‐4399. 25120824 PMC4129059

[10] Arber DA , Orazi A , Hasserjian R , et al. The 2016 revision to the World Health Organization classification of myeloid neoplasms and acute leukemia. Blood. 2016;127(20):2391‐2405. 27069254 10.1182/blood-2016-03-643544

[11] Li Y , Li Z , Lin H‐l , Chen X‐h , Li B . Primary cutaneous blastic plasmacytoid dendritic cell neoplasm without extracutaneous manifestation: case report and review of the literature. Pathology‐Research and Practice. 2011;207(1):55‐59. 20573459 10.1016/j.prp.2010.05.008

[12] Mehra S , Taylor J . Blastic plasmacytoid dendritic cell neoplasm: a comprehensive review of the disease, central nervous system presentations, and treatment strategies. Cells. 2024;13(3):243. 38334635 10.3390/cells13030243 PMC10854688

[13] Facchetti F , Vermi W , Santoro A , Vergoni F , Chilosi M , Doglioni C . Neoplasms derived from plasmacytoid monocytes/interferon‐producing cells: variability of CD56 and granzyme B expression. Am J Surg Pathol. 2003;27(11):1489‐1492. 14576486 10.1097/00000478-200311000-00015

[14] Tse E , Kwong Y‐L . The diagnosis and management of NK/T‐cell lymphomas. J Hematol Oncol. 2017;10(1):1‐13. 28410601 10.1186/s13045-017-0452-9 PMC5391564

[15] Tse E , Kwong Y‐L . NK/T‐cell lymphomas. Best Pract Res Clin Haematol. 2019;32(3):253‐261. 31585625 10.1016/j.beha.2019.06.005

[16] Pulitzer M . Merkel cell carcinoma. Surg Pathol Clin. 2017;10(2):399‐408. 28477888 10.1016/j.path.2017.01.013 PMC5443625

[17] Martín‐Martín L , López A , Vidriales B , et al. Classification and clinical behavior of blastic plasmacytoid dendritic cell neoplasms according to their maturation‐associated immunophenotypic profile. Oncotarget. 2015;6(22):19204‐19216. 26056082 10.18632/oncotarget.4146 PMC4662485

[18] Pagano L , Valentini CG , Pulsoni A , et al. Blastic plasmacytoid dendritic cell neoplasm with leukemic presentation: an Italian multicenter study. Haematologica. 2013;98(2):239‐246. 23065521 10.3324/haematol.2012.072645 PMC3561431

[19] Taylor J , Haddadin M , Upadhyay VA , et al. Multicenter analysis of outcomes in blastic plasmacytoid dendritic cell neoplasm offers a pretargeted therapy benchmark. Blood. 2019;134(8):678‐687. 31243042 10.1182/blood.2019001144 PMC6706810

[20] Bekkenk MW , Jansen PM , Meijer CJ , Willemze R . CD56+ hematological neoplasms presenting in the skin: a retrospective analysis of 23 new cases and 130 cases from the literature. Ann Oncol. 2004;15(7):1097‐1108. 15205205 10.1093/annonc/mdh268

[21] Roos‐Weil D , Dietrich S , Boumendil A , et al. Stem cell transplantation can provide durable disease control in blastic plasmacytoid dendritic cell neoplasm: a retrospective study from the European Group for Blood and Marrow Transplantation. Blood. 2013;121(3):440‐446. 23203822 10.1182/blood-2012-08-448613

